# Feasibility and Convergent Validity of an Activity Tracker for Low Back Pain Within a Clinical Study: Cross-sectional Study

**DOI:** 10.2196/18942

**Published:** 2021-03-26

**Authors:** Linda Xiaoqian Zhuo, Luciana Gazzi Macedo

**Affiliations:** 1 School of Occupational Therapy Faculty of Health Sciences Western University London, ON Canada; 2 School of Rehabilitation Science McMaster University Hamilton, ON Canada

**Keywords:** activity monitor, activity tracker, low back pain

## Abstract

**Background:**

Low back pain (LBP) is a highly prevalent condition affecting individuals of all ages. To manage the symptoms and prevent recurrences and flare-ups, physical activity in conjunction with self-management education is recommended. Tools such as diaries and questionnaires have been the gold standard for tracking physical activity in clinical studies. However, there are issues with consistency, accuracy, and recall with the use of these outcome measures. Given the growth of technology in today’s society, consumer-grade activity monitors have become a common and convenient method of recording physical activity data.

**Objective:**

The aim of this study is to test the feasibility and convergent validity of a Garmin Vivofit 3 activity tracker in evaluating physical activity levels in a clinical trial of patients with LBP.

**Methods:**

We recruited 17 individuals with nonspecific LBP referred from health care professionals or self-referred through advertisements in the community. The participants entered into a 12-week physical activity and self-management program. Physical activity was assessed using a self-reported questionnaire and the Garmin activity tracker. Activity tracker data (eg, steps taken, distance walked, and intensity minutes) were extracted weekly from the Garmin Connect online platform. Outcomes of pain and activity limitation were assessed weekly using a mobile app. A linear regression was conducted to evaluate if demographic factors (ie, age, gender, pain level) affected the adherence rates to the activity monitor. We also used Pearson correlations to evaluate the convergent validity of the Garmin activity tracker with the physical activity questionnaire.

**Results:**

The mean daily adherence rate for activity monitors was 70% (SD 31%) over the 26 weeks of study. The mean response rate for the weekly physical activity measures using REDCap for the first 12 weeks of the study was 91% (SD 17%). None of the hypothesized variables or questionnaires were predictors of response rate.

**Conclusions:**

The majority of participants were compliant with wearing the tracker, and demographic factors were not found to be predictors of adherence to wearing the device. However, there were poor correlations between the modified International Physical Activity Questionnaire Short Form (IPAQ-SF) and the activity monitor, demonstrating problems with convergent validity.

## Introduction

Low back pain (LBP) is the most common musculoskeletal condition worldwide [1]. Approximately 85% of individuals with LBP will be diagnosed with nonspecific LBP, which refers to pain not attributed to a specific diagnosis such as sciatica, ankylosing spondylitis, and vertebral fracture [2-4]. Considering the high prevalence of LBP, treatment and management of the condition remain an important area of investigation.

Recent studies suggest that lifestyle modification and adherence to physical activity are crucial to preventing recurrences or back pain flare-ups and improving one’s quality of life [5,6]. The majority of clinical practice guidelines recommend the use of education and exercise to manage LBP [7]. Exercise programs have shown to be successful at reducing pain, disability, and improving quality of life [8].

Levels of physical activity and exercise adherence are important outcome measures often used in clinical research. Traditional methods for tracking physical activity are diaries and questionnaires. However, these tools are unable to record real-time information and suffer from significant adherence problems and recall bias [9,10]. Studies that use diaries to track exercise patterns have lacked consistency, as participants often forget to make regular entries and frequently resort to recall for completing diaries post hoc [9]. It has been estimated that patients may fail to enter over 50% of requested physical activity data [9]. Questionnaires pose similar problems, where participants have a tendency to overestimate or underestimate values for question responses [10]. Factors such as day of the week when completing the questionnaire and self-esteem toward sensitive questions can influence the recall process [10]. Study participants often underestimate sitting duration up to 4.5 hours/day when solely relying on questionnaires to record the information post hoc [10]. Recent studies have shown that questionnaires have limited validity and reliability when collecting physical activity measures in the community [11]. Therefore, other stronger and more reliable tools may be needed for the collection of physical activity data within clinical studies.

Physical activity monitors such as pedometers and heart rate trackers have been used as alternatives for the collection of physical activity and exercise compliance data within clinical studies. Common measures that activity monitors can collect are steps taken per day, distance traveled per day, and intensity minutes obtained (amount of moderate and vigorous activities conducted). These activity trackers are exciting technologies that can collect and store a large number of data related to physical activity. These objective outcome measures evaluate physical activity and provide the opportunity to collect this information in the real world, during daily activities. Furthermore, activity monitors are less influenced by participant and evaluator bias; however, user reactivity to the activity monitor is a possibility, although the devices remain free of other biases. Research-grade activity trackers such as the ActiGraph are considered gold-standard tools for the collection of activity data [12]. Unfortunately, these trackers are costly, somewhat bothersome to wear, and require frequent uploads and charging, making its community use limited for collecting long-term data. Commercial-grade activity trackers represent an alternative for the collection of outcomes within studies [13,14]. Activity monitors such as Fitbit and Garmin devices tend to be more financially affordable, come in smaller and sleeker designs, possess a longer battery life (eg, up to 1 year), come in water-resistant forms, and can easily upload activity data to a mobile device [15,16]. These features make commercial-grade activity monitors appealing options, especially when compared with research-grade trackers that have shorter battery life and require more support for wear.

Commercial-grade activity trackers have been found to have some limitations in identifying low-intensity physical activity, particularly in older adults [12,17]. In addition, many activity trackers are worn on the wrist, which may present limitations in detecting activity from the lower limbs [18,19]. Clip-on activity trackers display similar issues in their limited ability to track movement from the entire body depending on their placement location. Although there are recognizable limitations for the use of commercial-grade activity trackers, the ease of using these activity trackers cannot be ignored when selecting outcome measures in research, especially when considering moderate and vigorous activities for which activity monitors have been found to have better validity [17]. They are economically priced for the public, employ user friendly systems, and can be easily worn on the wrist or clothing.

In addition to limitations related to the quality of the data collected, there are some concerns surrounding adherence rates in wearing and syncing activity monitors in clinical studies of long duration. Similar to diaries, participants are asked to wear activity monitors on a daily basis, charge the devices as appropriate, and sync with online platforms. To date, there is a gap in the literature on the feasibility of using a commercial-grade Garmin activity tracker in clinical studies. Understanding its value and usage in clinical trials can be beneficial in paving the way to more practical applications of commercial-grade products in scientific research. Thus, the primary objective of this study was to evaluate the feasibility of using a Garmin Vivofit 3, a consumer-grade activity tracker, to collect data in a clinical trial of patients with LBP. Feasibility will be measured in terms of adherence rates in wearing the monitor and whether there are differences in age, gender, or pain level in adherence rates. The secondary objective was to evaluate the convergent validity of the Garmin Vivofit 3 with the items of the modified International Physical Activity Questionnaire Short Form (IPAQ-SF) questionnaire.

We hypothesized that women and younger participants with higher levels of pain would be more likely to wear and sync their activity monitors. We also hypothesized that there would be a moderate correlation (Pearson correlation >0.6) of physical activity data with physical activity information collected using the IPAQ-SF.

## Methods

### Study Design

This is a project imbedded into a pretest posttest parent study aiming to evaluate the feasibility of a community-based physical activity program for patients with nonspecific LBP. The primary goal of the parent study was to observe whether the program could prevent recurrences of flare-ups of LBP and mitigate the negative consequences of the condition. The STROBE checklist used to report epidemiological studies was used in this report [20]. The study received ethical approval from the Hamilton Integrated Research Ethics Board (#2721) on June 15, 2017, and all participants signed a consent form prior to inclusion.

### Recruitment for Parent Study

Participants were recruited to participate in the clinical trial from local physiotherapists, chiropractors, physicians, and community advertisements in Hamilton, Ontario, Canada. Participants were included if they met the following inclusion criteria: (1) discharged <3 months from physiotherapy, chiropractic, or osteopathic care following a course of treatment for LBP; (2) have nonspecific LBP; and (3) between 18 and 80 years of age. Participants were excluded if they met any of the following criteria: (1) ongoing high pain intensity, defined as pain intensity of 6 or more on a 0-10-point scale. The cut off of 6/10 is used in the literature to dichotomize low to high pain intensity [21,22]; (2) comorbidity preventing participation in physical activity as evaluated by the Physical Activity Readiness Questionnaire (PAR-Q) from the American College of Sports Medicine guidelines [23]; (3) inadequate English to complete outcome measures; (4) currently participating in an exercise program similar to the one we will evaluate; and (5) history of spine surgery.

### Equipment

The Garmin Vivofit 3 is a commercial-grade activity monitor that that tracks steps taken, calories, distance traveled, intensity minutes, and sleep. It features a 1-year battery life, enabling it to track one’s activity 24/7. The monitor is able to sync with the online Garmin Connect platform to provide further details of one’s activities and connect with other users [16].

REDCap was the software used to create and send questionnaires to participants’ emails as well as record their responses.

### Procedures

Each individual participated in an initial appointment during which the research assistant gave an overview of the study, and participants signed consent forms. Baseline questionnaires were completed on the REDCap platform through a link sent to the participant’s email address. Longitudinal data collection procedures were explained to the participant and their smartphones were set up to receive study notifications for weekly pain data collection through the MetricWire app. Garmin Vivofit 3 activity monitors were distributed to all participants and the research assistant instructed them on how to sync their tracker with a smartphone device or a computer. Participants were instructed to wear the activity monitor on a daily basis and only remove during swimming or showers. All participants underwent a 12-week exercise and education program and received 4 months free membership at a local YMCA gym.

### Outcome Measures

Pain (Numerical Rating Scale), function (Patient-Specific Functional Scale), disability (Roland Morris Disability Questionnaire), health-related quality of life (EQ-5D-5L), and physical activity (activity tracker and modified IPAQ) levels were collected at baseline, at the end of the 12-week intervention, and at 6 months’ follow-up. In addition, pain, disability, and mood outcomes and physical activity data were collected once a week for 26 weeks. Pain outcomes were collected once a week using a smartphone app that produced weekly notifications. All participants were asked to wear an activity monitor for the duration of 26 weeks and sync their devices biweekly with an online platform. One of the study investigators (LZ) logged into the Garmin website and extracted physical activity data for all participants. The activity monitor data extracted were steps taken per day, distance traveled per day, and intensity minutes obtained per day. Finally, once a week for the duration of the interventions (12 weeks), participants received a REDCap link to complete a self-management action plan and completed the IPAQ-SF from which responses about moderate, vigorous activity, and walking were extracted.

### Statistical Analysis

Descriptive statistics of the population including age, sex, education level, pain, function, disability, and quality of life outcomes were presented as mean (SD) or n (%) when appropriate.

#### Response Rates

Response rates were calculated for the 12-week period of the intervention as well as for the 26 weeks of the study (intervention + follow-up period). Weekly adherence rates with wearing the monitor, how often participants synced their data to the mobile app, and how often researchers needed to send reminders regarding syncing were presented. Univariate linear regression and a multiple regression using backward elimination were used to identify whether age, gender, and baseline pain, function, and disability predict the number of times a participant “adhered” to the activity monitor protocol (wear and sync). A Bonferroni correction for multiple comparisons using an α level divided by the number of predictors (n=9) was conducted with α=.006.

#### Convergent Validity

A Pearson correlation was used to investigate the association between physical activity level reported weekly on the IPAQ-SF (self-reported amount of time per day spent engaging in moderate, vigorous physical activity, and walking) and the activity monitor (step counts, distance traveled, and number of intensity minutes). A 1-tailed hypothesis testing comparing the identified Pearson correlation with the expected null hypothesis of 0.6 was conducted. STATA (version 14.0; StataCorp) was used for all statistical analyses.

## Results

**Figure 1:**
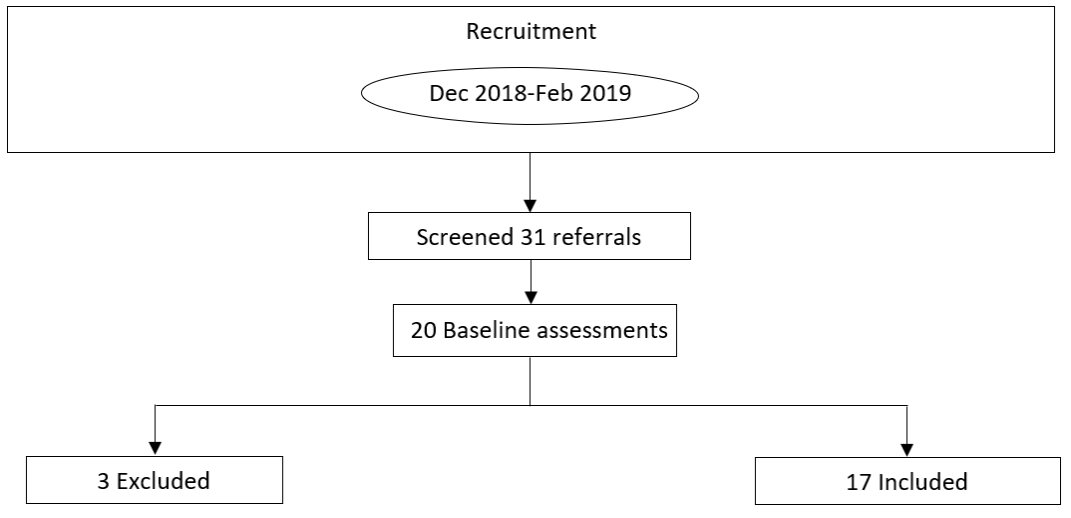
Schematic representation of study timeline.

Participants in this study were either referred from physiotherapists and chiropractors working in the Hamilton community or recruited from advertisements through the Les Chater YMCA’s social media. A total of 21 individuals were referred to the study by health care professionals, and 10 participants contacted the investigators following a social media advertisement through the YMCA from December 2018 to February 2019. Of those 31, 20 individuals were deemed eligible to participate, and 17 were ultimately included. A lack of time was the justification provided by all 3 eligible participants who did not agree to participate in the study. A schematic of the study timeline can be found in Figure 1. A list of patient demographic information can be found in Table 1.

**Table 1:** Participant demographic characteristics and questionnaire scores (N=17).

Variable			Value	
**Sex, n (%)**				
	Female	9 (53)		
	Male	8 (47)		
**Marital status, n (%)**				
	Married	14 (82)		
	Divorced	1 (6)		
	Common Law	1 (6)		
	Single	1 (6)		
**Occupation, n (%)**				
	Not working^a^	4 (24)		
	Working	13 (76)		
**Employment, n (%)**				
	Full-time full duties	8 (47)		
	Full-time selective duties	1 (6)		
	Part-time full duties	2 (12)		
	Part-time selective duties	2 (12)		
	Not seeking employment	4 (24)		
**Smoking/medication, n (%)**				
	Smoking	1 (6)		
	Taking painkillers	2 (12)		
**Physical activity level, n (%)**				
	Moderate physical activity^b^	10 (59)		
**Education, n (%)**				
	High-school diploma	2 (12)		
	Diploma	5 (29)		
	Bachelor’s degree	1 (6)		
	Postgraduate degree	3 (18)		
	Other	6 (35)		
**Characteristic, mean (SD)**				
	Age (years)	54.9 (11.7)		
	Weight (kg)	82.9 (18.5)		
	Height (cm)	175.1 (10.2)		
	BMI (kg/m^2^)	26.9 (4.8)		
	Duration of low back pain (months)	62.9 (69.7)		
**Scale, mean (SD)**				
	Weekly pain rating	4.9 (2.5)		
	Patient-Specific Functional Scale	5.7 (2.5)		
	Roland Morris Disability Questionnaire	6.2 (4.)		
	Pain Self-Efficacy Questionnaire	48.5 (11.2)		
	Coping Strategies Questionnaire	11.9 (6.)		

^a^At the start of the study, 3 participants were not working prior to their low back pain. Currently, 4 people are not working.

^b^At least 30 minutes of activity per day, 3 times a week.

### Response Rates

**Figure 2:**
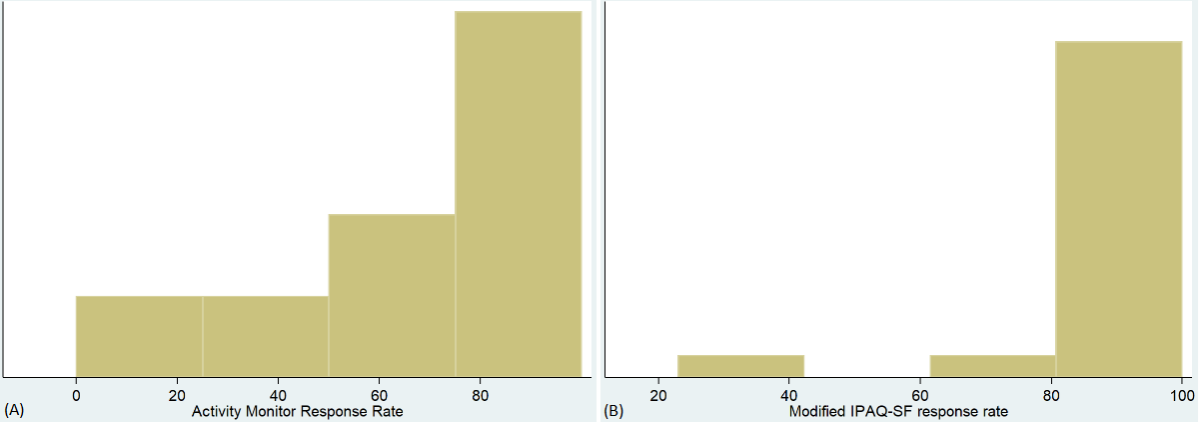
Histogram of adherence rate and response rate to (A) activity monitor (B) modified IPAQ. IPAQ-SF: International Physical Activity Questionnaire Short Form.

The mean adherence rate (wearing and syncing) for activity monitors was 128 out of 182 (70%; SD 31%) total days, with a median of 141 days (77%; IQR 47%) over the 26 weeks of the study. Average response rate for the IPAQ-SF, which was collected during the first 12 weeks of the study, was 11 times (92%; SD 17%), with a median of 12 (100%; IQR 8%). Adherence rate of the activity monitors was highly skewed, as demonstrated by the histogram (Figure 2). There was 1 participant that did not respond to any of the activity measures or wear the activity monitor. In addition, there was 1 participant that lost the activity monitor and was thus unable to continue syncing. The participant with 0 weeks of data experienced log-in issues associated with his Garmin account. There were no significant differences in baseline characteristics between the compliant and noncompliant group of participants (*P*<.05 for all cases; see Table 2).

Univariate linear regression demonstrated that none of our hypothesized variables or questionnaires were predictors of response rate (Table 2). However, given the small sample size of this study, we were underpowered to identify significant associations. We were unable to build a multivariate model using our correct α level of .006. However, these results are likely due to a type II error.

**Table 2:** Univariate linear regression analysis of participant characteristics and questionnaire responses as predictors of activity monitor response rate.

Predictor	Regression coefficient (95% CI)	*P* value	*R*^2^ (%)
Age	–0.5 (–1.9 to 0.9)	.46	4
Gender	23.6 (–7.8 to 55.1)	.13	15
Education	4.4 (–5.3 to 14.1)	.35	8
Pain duration	–0.02 (–0.3 to 0.2)	.89	0.2
Weekly pain rating	5.8 (–0.1 to 11.7)	.05	23
Patient-Specific Functional Scale	4.1 (–2.4 to 10.5)	.20	11
Roland Morris Disability Questionnaire	–0.9 (–4.7 to 2.9)	.62	0.2
Pain Self-Efficacy Questionnaire	1.0 (–0.3 to 2.3)	.14	14
Coping Strategies Questionnaire	–2.1 (–4.5 to 0.2)	.08	20

### Convergent Validity

Convergent validity was calculated for all data collected within the first 12 weeks of the intervention, meaning that 204 weeks of data were incorporated into the analysis (17 participants × 12 weeks). On the self-reported physical activity questionnaire, participants were asked to recall the amount of physical activity that they performed (vigorous, moderate, or walking) on an average day during the week, and thus, data represent minutes per day. Likewise, activity data such as step count, distance traveled (miles), and intensity minutes were entered into the analysis as averages per week, thus representing steps, miles, or minutes on an average day, respectively. If no data were collected for a specific day on the activity monitor (ie, 0 steps), then this data line was excluded from the calculations. Pearson correlation was poor and did not reach the threshold for validity in any of the outcomes (Table 3).

To further evaluate where inconsistencies may exist between physical activity data and the IPAQ-SF, individual patient data were observed (Table 3). The observed data demonstrated a variability in responses with some patients that underestimated self-reported activities while others overestimated self-reported activities. The results demonstrated poor correlations between the data collected from the activity monitor and the responses from the IPAQ-SF with correlations not statistically greater than the hypothesized *r*=0.6.

**Table 3:** Pearson correlation of average activity monitor data and IPAQ-SF^a^ responses.

Measurement tool			Garmin							IPAQ				
			Step count		Distance traveled (miles)		Intensity (minutes)		Vigorous activity (minutes)			Moderate activity (minutes)		Walking (minutes)
**Garmin**														
	Step count	1.000												
	Distance traveled (miles)	0.99^b^		1.000										
	Intensity minutes	0.76		0.77		1.0000								
**IPAQ**														
	Vigorous activity (minutes)	0.32		0.31		0.24		1.0000						
	Moderate activity (minutes)	0.32		0.30		0.29		0.82^b^			1.0000			
	Walking (minutes)	0.10		0.08		0.04		0.24			0.39		1.0000	

^a^IPAQ-SF: International Physical Activity Questionnaire Short Form.

^b^Denotes correlation is statistically significantly greater than the hypothesized *r* value of 0.6.

## Discussion

### Principal Results

The results of this study indicated that the mean adherence rate for wearing and syncing activity monitors was 70% (128/182) at 26 weeks, with an average response rate of 92% (11/12) for the IPAQ-SF collected using the REDCap survey. Other studies have found similar levels of engagement [24,25]. There were no variables that predicted response rate as per our univariate models. Given the poor adherence rates of self-reported physical activity questionnaires in the long term (eg, diaries), activity monitors represent a good alternative with moderate to high levels of compliance as illustrated in this study. This is especially true if some of the issues, such as replacing lost activity monitors and solving log-in errors to the online platform, can be addressed.

The correlation between the physical activity reported from the activity monitor and self-reported measures from the IPAQ-SF was poor and did not reach the threshold necessary for validity (*r*=0.6), thus indicating poor convergent validity between the 2 constructs. However, it is important to note that physical activity questionnaires suffer from overestimations and underestimations, which limit their ability to act as a comparison for the validity of activity monitor data.

### Adherence Rates

Existing literature that employed tools such as pedometers, smartphone apps, and SMS text messages present a variety of findings on the relationship between participant demographic factors and adherence rates.

#### Age

Within our study, there was a wide age range among participants (18-80 years) but there was no difference for response rate among the ages, potentially due to the small sample size and a lack of power. However, it is interesting to note that all of the noncompliant individuals and those that experienced difficulties with the activity monitor were part of an older demographic group (>50 years). Other studies have identified different age groups with higher adherence rates with activity monitors, SMS text messages, or smartphone apps. In accordance with an article reporting on Australian adolescents, it was previously noted that there is low compliance among the participants in the study due to discomfort of wearing activity devices, the risk of receiving unwanted attention, and feeling embarrassed [26]. Similarly, another study of patients with LBP indicated that participants who withdrew from SMS text message studies were usually younger in age [27]. By contrast, a study concerning the use of medical apps by physicians for patient care demonstrated younger individuals using the app more than older individuals [28]. Similarly, younger individuals were reported to be more likely to use a wearable activity monitor in a US national physical activity survey [29]. The differing results in adherence rates among age groups may reflect the type of data collection tool used and preferences between age groups for a specific tool.

#### Gender

There has been no consensus thus far on whether males or females are more likely to adhere with using new methods of data collection such as activity monitors and smartphone apps. In this study there was no evidence of gender impacting response rates. A diabetes-related study requested participants to wear an Actical (Philips Respironics) accelerometer for a week to investigate diabetes, pulmonary, and cardiovascular disease risk factors, as well as morbidity and mortality. Results indicated that male participants demonstrated higher adherence rates [25]. However, a study on Swedish, Dutch, and Danish populations including patients with LBP in primary care reported that those who dropped out from studies that used SMS text messages were typically male [30-32]. The disagreement in the literature indicates that there is a lack of information and controversial results on the role of gender in adherence rates.

#### Levels of Pain

Participant’s pain levels were not found to predict adherence to wear for the activity monitor used in this study. In line with our findings, pain levels were not predictors of response rate to an SMS text messaging system used to collect outcome measures within a study of LBP [33]. The poor correlation between adherence and pain levels in these 2 studies may be due to the ease of wearing the Garmin monitor or one’s instinctive ability to send SMS text messages [33].

### Correlation With IPAQ-SF

The Garmin Vivofit 3 activity monitor collected objective physical activity data in the form of step counts, distance traveled, and intensity minutes. These measures were collected in real time on an ongoing basis, with weekly averages calculated by the device. The IPAQ-SF was used to record subjective estimations of the participant’s physical activity patterns over the span of a week. It specifically inquired about the number of days spent doing moderate and vigorous activity, as well as the amount of time spent during one of those days. The responses from the modified IPAQ-SF used in the analysis of our study were estimations of the number of hours of an activity an individual performed in 1 day on a particular week. However, the Garmin activity monitor provided daily measures of activity. Thus, to compare with an estimated average hour per day as presented on the IPAQ-SF, the activity monitor data entered in our validity analysis were averaged per week to represent daily averages. Pearson correlations were used to evaluate the convergent validity of the physical activity data from the Garmin Vivofit 3 in comparison with the IPAQ-SF. We were unable to find any studies that compared the IPAQ-SF with the same variables from a Garmin activity monitor. Of the studies that conducted comparisons between other activity monitors and the IPAQ, the findings presented weak correlations [34]. For example, in a study investigating the validity of the IPAQ-SF in measuring physical activity of patients with chronic fatigue syndrome, the Actical accelerometer measure of vigorous activity was identified to have weak correlations with the IPAQ-SF self-reported measure of vigorous activity [35]. These results are in line with our study, suggesting discrepancies between activity measures and the IPAQ. The weak correlations between the 2 may be the result of the Garmin tracker’s inability to detect small-scale changes in activity, the recall bias in completing the IPAQ-SF post hoc, or a combination of these.

### Limitations

Limitations to the study included a small sample size and the inclusion of patients with no smartphone or a tablet. Our sample size of 17 individuals did not provide enough statistical power to make definite conclusions about the analysis conducted. In addition, most people were compliant, and thus variety in response rates were low, also compromising some of our comparison’s power. The IPAQ-SF was designed to ask participants about their physical activity levels averaging 1 day per week instead of collecting daily measures. This made it difficult to design accurate comparisons with the physical activity data collected by the Garmin tracker. Another limitation pertains to the inclusion of a participant in the study who did not have access to a smartphone or tablet with Bluetooth. This presented issues with Bluetooth syncing to the mobile app. Possession of a smartphone was not one of the criteria for inclusion into the study, thus the individual was enrolled into the study. To accommodate the lack of a mobile device, this participant was provided with an ANT stick to sync her activity monitor to a computer. However, collecting data from this individual was not ideal as her methods of syncing (the frequency of which was used as a measure of compliance) differed from that of the other participants. Finally, we did not collect information on the specific daily wear time for the activity monitors, which means participants could have used and synced information that was collected on a short period during the day rather than all day as expected.

### Implications and Future Directions

With a constantly aging population and a high prevalence of the disease, LBP rates will continue to rise and require continuous health care resources. Moving forward, the results from this pilot study may be used to guide future studies and grant applications. Subsequent studies should use this information to develop strategies to boost adherence in older adults with longer back pain duration and poorer self-efficacy. The poor convergent validity between the IPAQ-SF and the Garmin Vivofit 3 raises questions about the validity of these measures in assessing physical activity. Other possible methods include using a commercial-grade activity monitor in combination with a physical activity diary as a more feasible method of tracking physical activity. Future studies could potentially use a research-grade activity monitor such as the ActiGraph to obtain more accurate measures of physical activity. Despite issues with validity, the majority of participants were compliant with wearing the tracker, and thus activity monitors may still be a useful tool in scientific research if used in combination with other measures.

## Abbreviations

IPAQ-SF: International Physical Activity Questionnaire Short Form
LBP: Low back pain
PAR-Q: Physical Activity Readiness Questionnaire
